# Supplementary figures and images for: Characterization of constitutive ER-phagy of excess membrane proteins
Source: PLoS Genet. 2020 Dec 4;16(12):e1009255. doi: 10.1371/journal.pgen.1009255 (PMC7744050; doi:10.1371/journal.pgen.1009255)

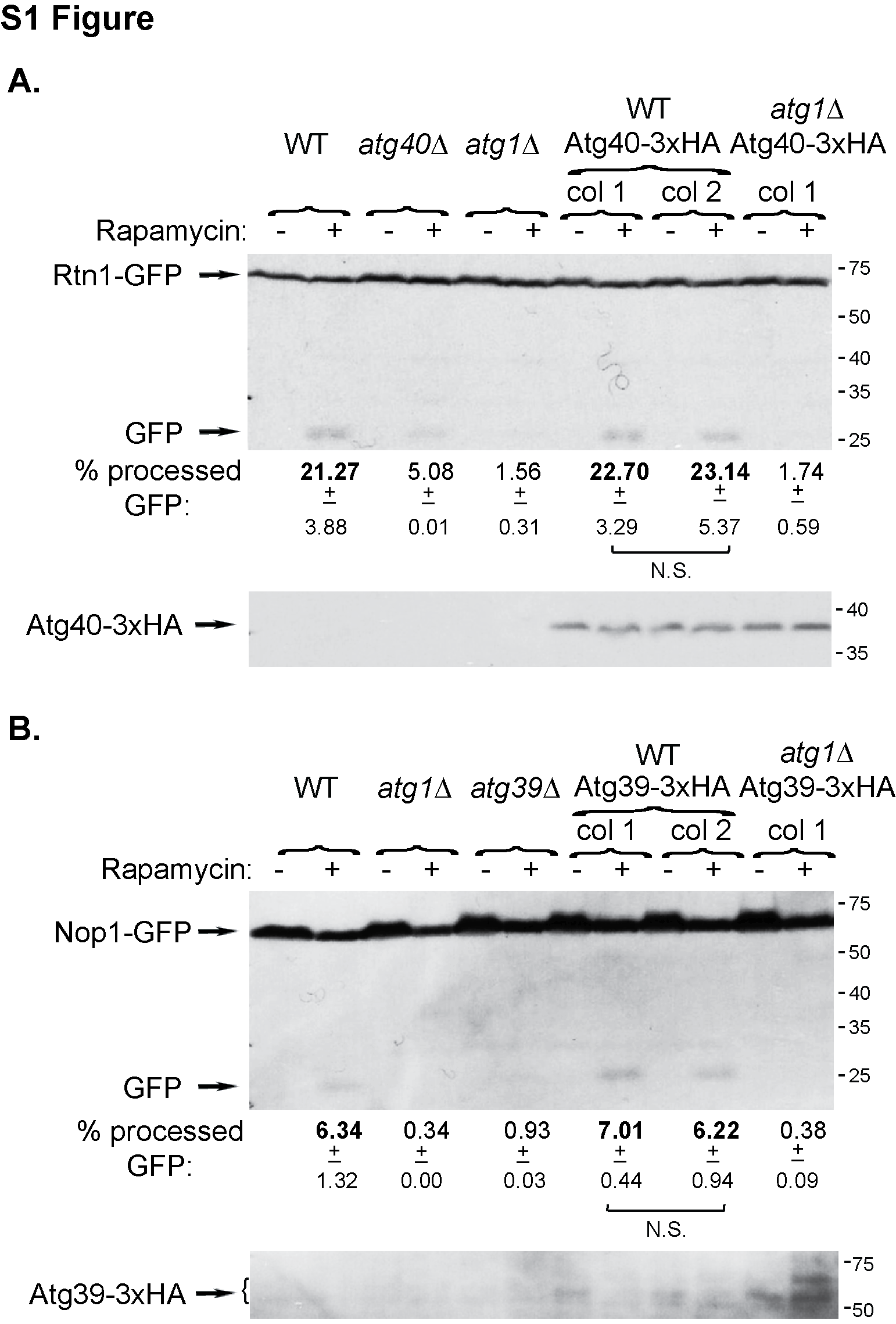

Supplement: S1 Fig — A. Atg40-3xHA can shuttle its Rtn1-GFP ER cargo for degradation under rapamycin-induced stress. Wild type and atg1Δ mutant cells expressing endogenously tagged Atg40-3xHA were transformed with a CEN plasmid expressing Rtn1-GFP under its own promoter. Cells were grown in normal medium (YPD) or under stress (+Rapamycin) and processing of Rtn1-GFP to GFP was determined by immuno-blot using anti-GFP antibodies. From left to right: WT, atg40Δ, atg1Δ, WT expressing Atg40-3xHA (two colonies), and atg1Δ expressing Atg40-3xHA. Shown from top to bottom: strain, growth conditions (- or + rapamycin), GFP blot, quantification of the % processed GFP, +/- STD, p-value, and HA blot (to confirm expression of Atg40-3xHA). >20% of the Rtn1-GFP is processed to GFP under stress in WT, but not atg40Δ or atg1Δ mutant cells. Importantly, cells expressing Atg40-3xHA as the only copy process Rtn1-GFP as well as WT cells (but not in atg1Δ cells). B. Atg39-3xHA can shuttle its Nop1-GFP ER cargo for degradation under stress. The same experiment described in panel A, was done with cells expressing endogenously tagged Atg39-3xHA and transformed with a CEN plasmid expressing Nop1-GFP under its own promoter. ~6–7% of the Nop1-GFP is processed to GFP under stress in WT, but not atg39Δ or atg1Δ mutant cells. Importantly, cells expressing Atg39-HA (bands within the bracket [15]) as the only copy, process Nop1-GFP as well as WT cells (but not in atg1Δ cells). Note: the level of Atg39-HA is >25-fold lower when cells are grown in YPD (as was done in this experiment that did not require selection of a plasmid) than in SD [55]. Results in this figure represent 3 independent experiments. (TIF) [file pgen.1009255.s001.tif]

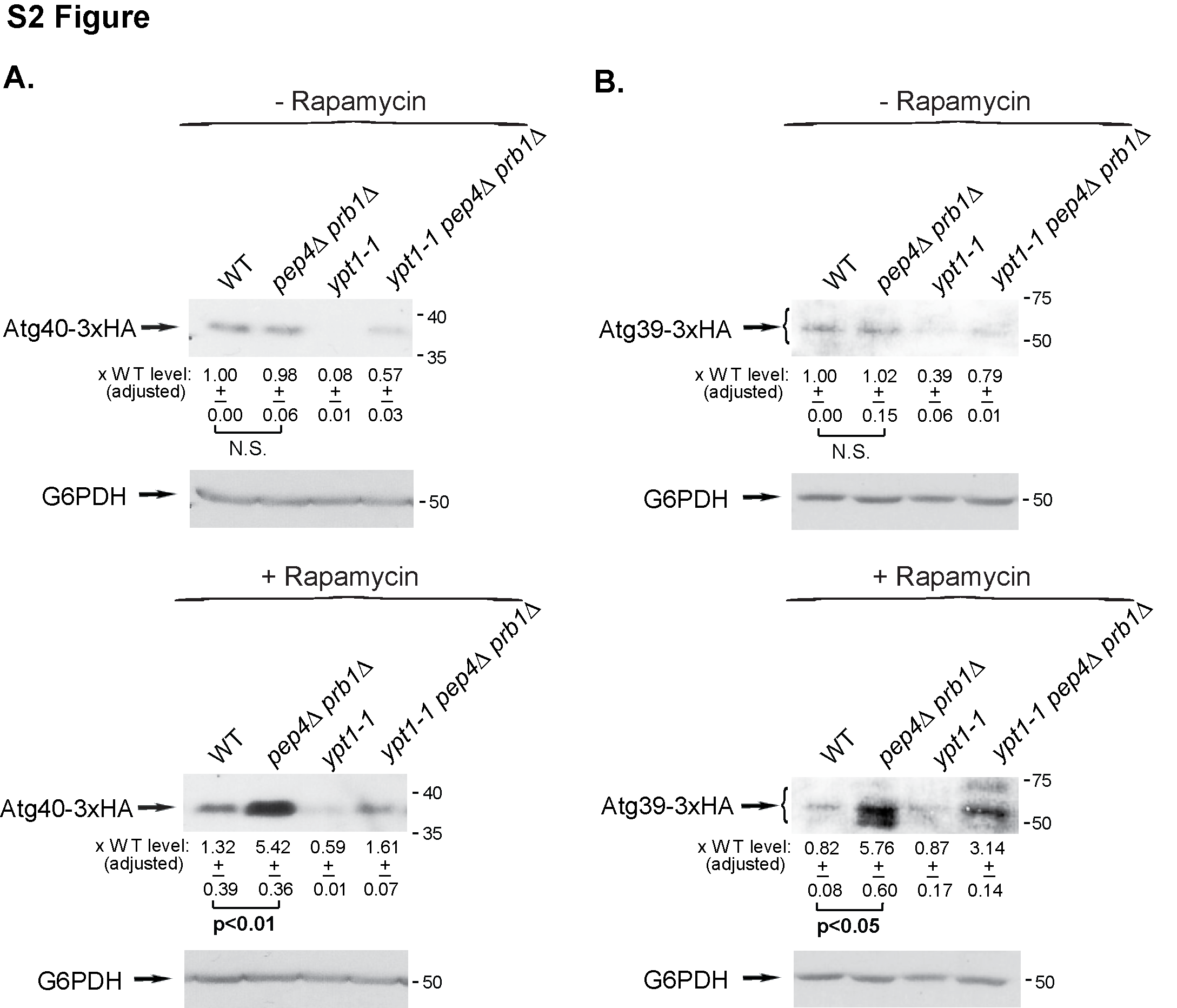

Supplement: S2 Fig — Endogenous Atg40 (A) and Atg39 (B) were tagged with 3xHA at their C-termini in four strains, WT, pep4Δ prb1Δ, ypt1-1, and ypt1-1 pep4Δ prb1Δ. Cells were grown in normal medium (SD-Rapamycin, top) or under stress (SD+Rapamycin, bottom). The level of Atg40-3xHA and Atg39-3xHA in cell lysates was determined using anti-HA antibodies and immuno-blot analysis (G6PDH was used as a loading control). Shown from top to bottom: growth conditions, strain, HA blot, quantification of the HA-tagged protein band: fold over wt, +/- STD, p value, and G6PDH blot. Under stress (+Rapamycin), the levels of both Atg40-3xHA and Atg39-3xHA (bands within the bracket [15]) increases by ~5-folds in cell defective in vacuolar proteolysis (pep4Δ prb1Δ), showing that they are delivered for degradation to the vacuole. The levels of Atg40 and Atg39 are lower in ypt1-1 mutant cells during normal growth and they increase during nutritional stress in proteolysis defective cells. Results from in this figure represent 3 independent experiments. (TIF) [file pgen.1009255.s002.tif]

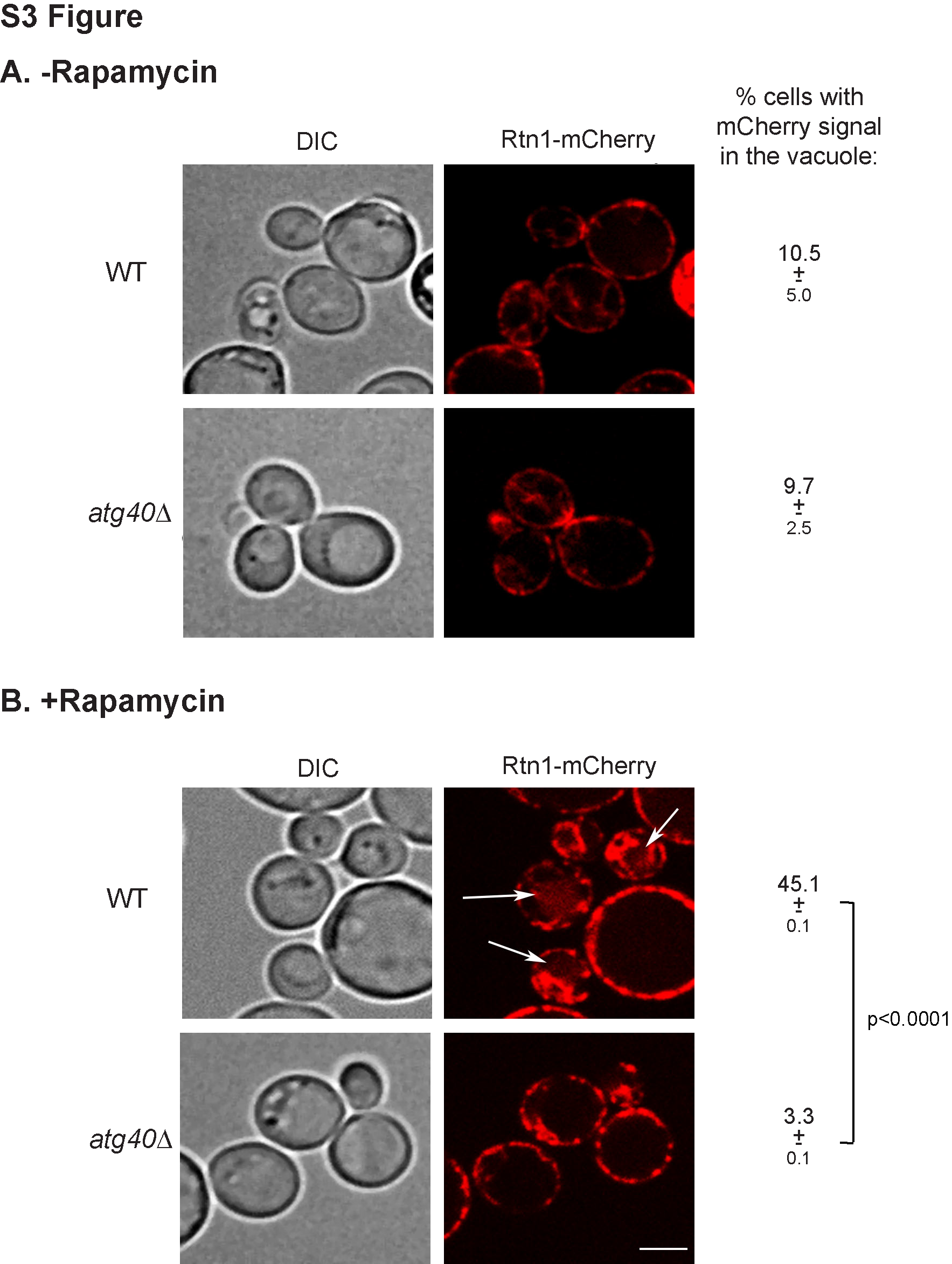

Supplement: S3 Fig — Endogenous Rtn1 was tagged with mCherry at its C-terminus (as in Fig 4C) in wild type and atg40Δ mutant cells. Cells were grown to mid log (A) and treated with rapamycin for 16 hours (B), were visualized by live-cell fluorescence microscopy. Shown from left to right: DIC, mCherry, % cells with Rtn1-mCherry in the vacuole; +/-, STD, and p value. Rtn1-mCherry localizes to the ER of both wild type and atg40Δ mutant cells during normal growth. Under stress (+rapamycin), it is delivered to the vacuole in 45% of wild type, but not atg40Δ mutant, cells. >150 cells were visualized for each data point; arrows point to Rtn1-mCherry in the vacuole; size bar, 1μ. Results in this figure represent 4 independent experiments. (TIF) [file pgen.1009255.s003.tif]

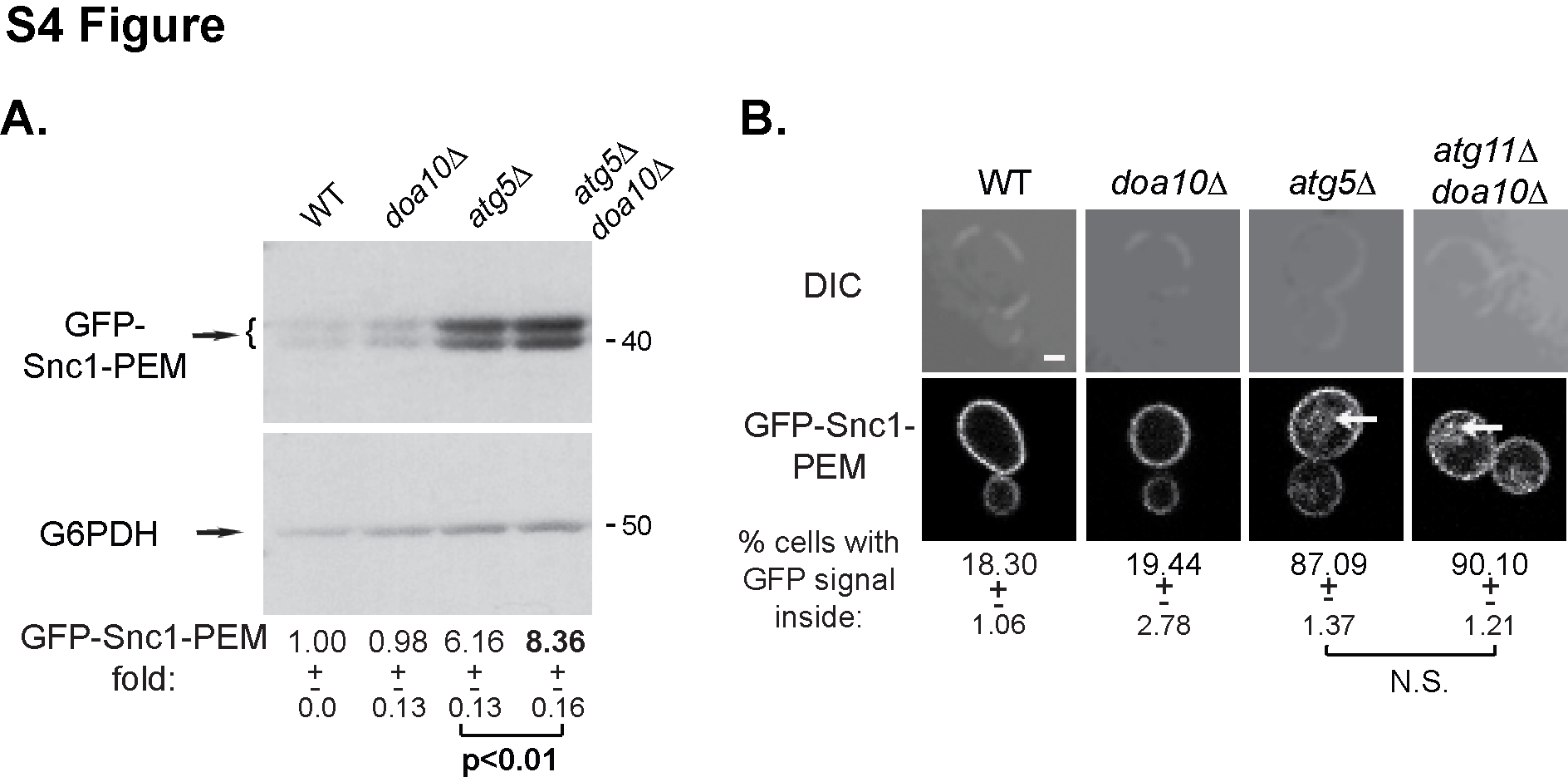

Supplement: S4 Fig — WT and indicated mutant cells overexpressing GFP-Snc1-PEM were grown in normal growth medium (SD+N). The level of GFP-Snc1-PEM in cell lysates was determined by immuno-blot analysis using anti-GFP antibodies (A); intracellular accumulation of GFP-Snc1-PEM was determined by live-cell fluorescence microscopy (B). Results are presented as in Fig 2. A 35% increase in the level of GFP-Snc1-PEM is observed when doa10Δ is combined with atg5Δ. Results in this figure represent 3 independent experiments. (TIF) [file pgen.1009255.s004.tif]

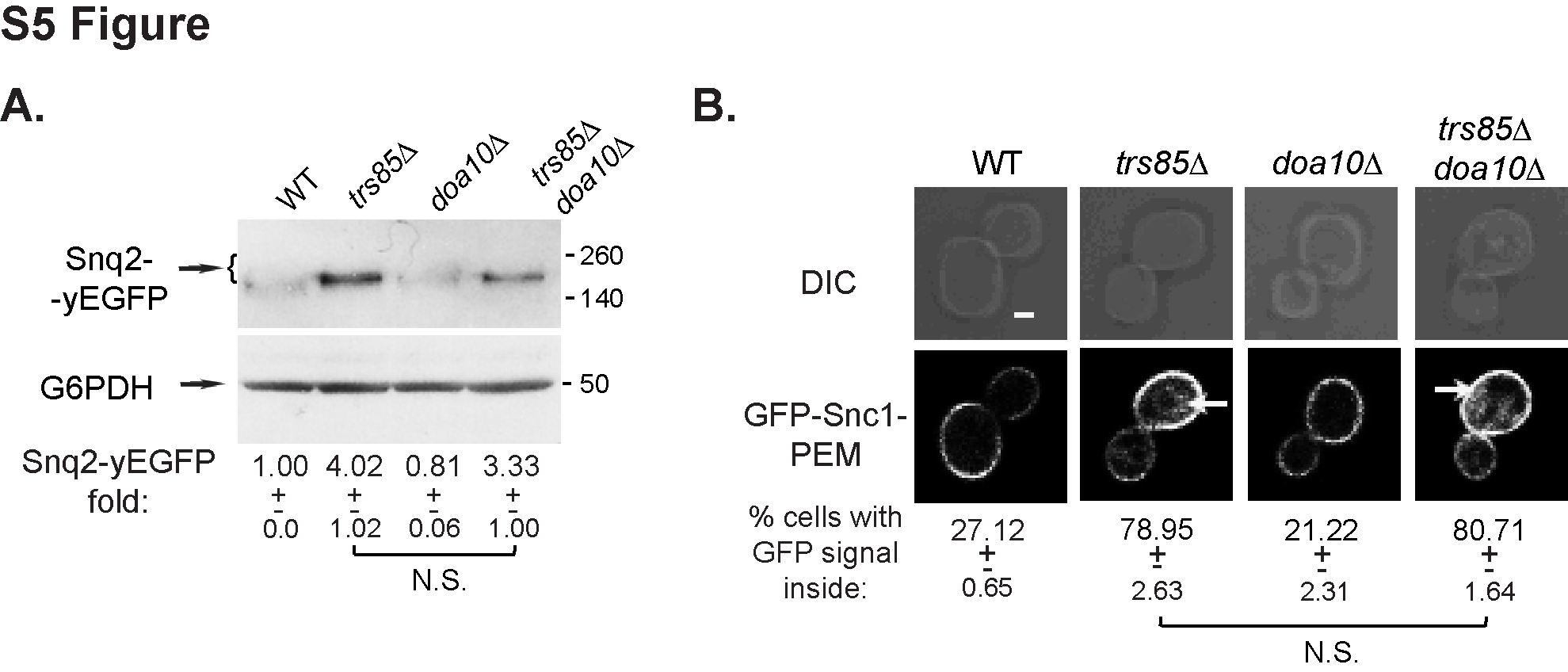

Supplement: S5 Fig — WT and indicated mutant cells overexpressing Snq2-yEGFP were grown in normal growth medium (SD+N). The level of Snq2-yEGFP in cell lysates was determined by immuno-blot analysis using anti-GFP antibodies (A); intracellular accumulation of Snq2-yEGFP was determined by live-cell fluorescence microscopy (B). Results are presented as in Fig 2. No increase in the level of Snq2-GFP is observed in doa10Δ or when it is combined with trs85Δ. Results in this figure represent 3 independent experiments. (TIF) [file pgen.1009255.s005.tif]

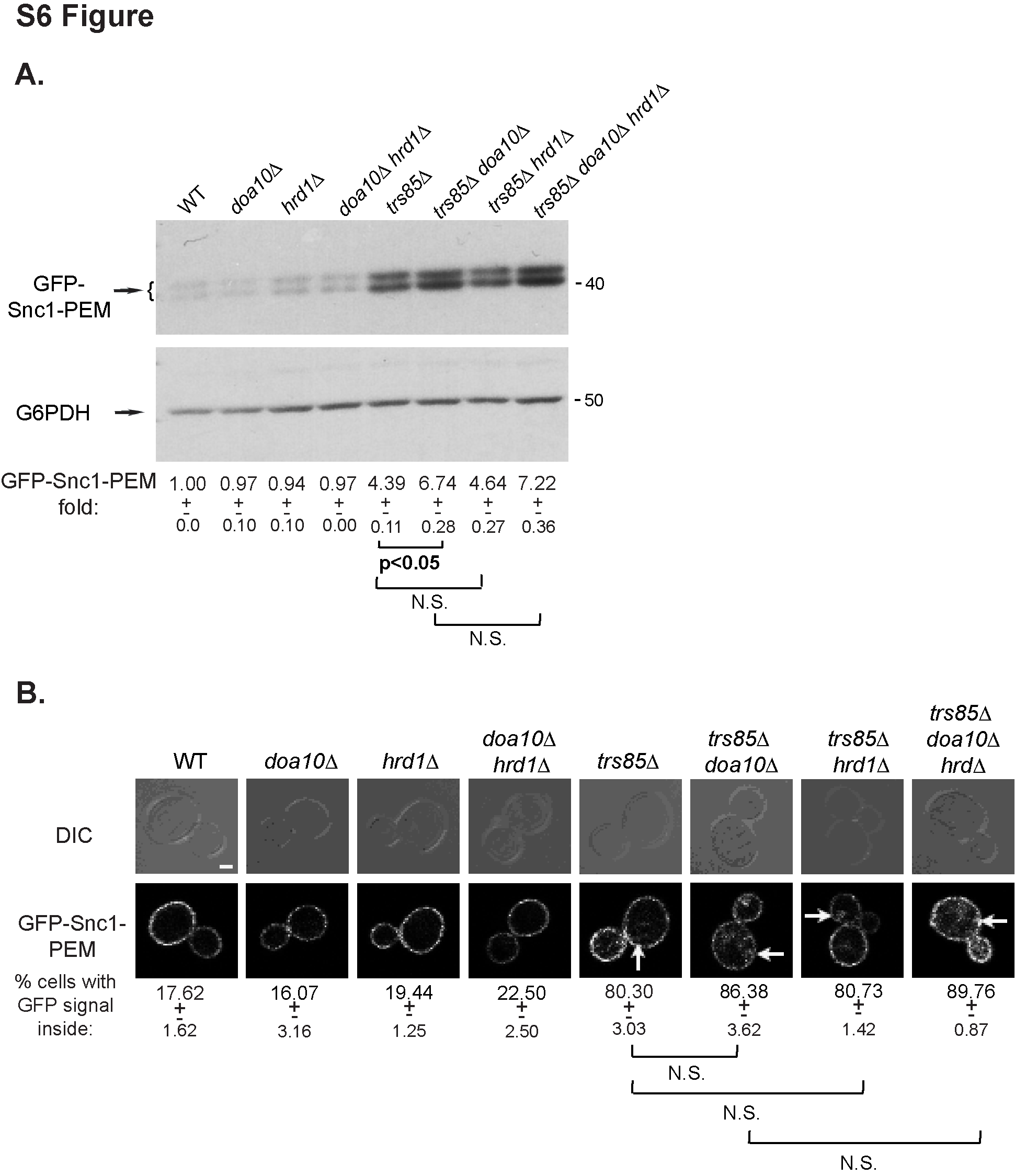

Supplement: S6 Fig — WT and indicated mutant cells overexpressing GFP-Snc1-PEM were grown in normal growth medium (SD+N). The level of GFP-Snc1-PEM in cell lysates was determined by immuno-blot analysis using anti-GFP antibodies (A); intracellular accumulation of GFP-Snc1-PEM was determined by live-cell fluorescence microscopy (B). Results are presented as in Fig 2, Whereas doa10Δ results in 50% increase of GFP-Snc1-PEM when combined with trs85Δ, no increase in the level of GFP-Snc1-PEM is observed in hrd1Δ, or when it is combined with trs85Δ, doa10 or trs85Δ doa10. Results in this figure represent 2 independent experiments. (TIF) [file pgen.1009255.s006.tif]
