# Supplementary material for: Characterization of constitutive ER-phagy of excess membrane proteins
Source: PLoS Genet. 2020 Dec 4;16(12):e1009255. doi: 10.1371/journal.pgen.1009255 (PMC7744050; doi:10.1371/journal.pgen.1009255)
Supplement: S1 Table — (DOCX) [file pgen.1009255.s007.docx]

**S1 Table. Yeast strains used in this study.**

| **Strain** | **Alias** | **Genotype** | **Source** |
| --- | --- | --- | --- |
| NSY825 | BY4741 | *Mat****a*** *leu2Δ0 ura3Δ0 his3Δ1 met15Δ0* | (Brachmann et al., 1998) |
| NSY1510 | *atg11Δ* | NSY825 *atg11Δ::KAN* | (Lipatova et al., 2012) |
| NSY1893 | *atg5Δ* | NSY825 *atg5Δ::KAN* | This study |
| NSY1894 | *atg7Δ* | NSY825 *atg7Δ::KAN* | This study |
| NSY1711 | *atg17Δ* | NSY825 *atg17∆::KAN* | (Lipatova and Segev, 2015) |
| NSY1712 | *atg11Δ atg17∆* | NSY1510 *atg17∆::HYGRO* | (Lipatova and Segev, 2015) |
| NSY2000 | *atg39Δ* | NSY825 *atg39Δ::KAN* | This study |
| NSY2001 | *atg40Δ* | NSY825 *atg40Δ::KAN* | This study |
| NSY2004 | *atg39Δ atg40∆* | NSY2001 *atg39Δ::NAT* | This study |
| NSY2002 | *atg11Δ atg39∆ atg40Δ* | NSY1510 *atg40Δ::HYGRO atg39Δ::NAT* | This study |
| NSY2003 | *atg17Δ atg39Δ atg40Δ* | NSY1711 *atg40Δ::HYGRO atg39Δ::NAT* | This study |
| NSY2005 | *ATG40-3xHA* | NSY128 *ATG40-3xHA::KAN* | This study |
| NSY2006 | *ypt1-1 ATG40-3xHA* | NSY55 *ATG40-3xHA::KAN* | This study |
| NSY2007 | *ATG40-3xHA pep4∆ prb1∆* | NSY1676 *ATG40-3xHA::KAN* | This study |
| NSY2008 | *ypt1-1 ATG40-3xHA pep4∆ prb1∆* | NSY1678 *ATG40-3xHA::KAN* | This study |
| NSY2009 | *ATG39-3xHA* | NSY128 *ATG39-3xHA::KAN* | This study |
| NSY2010 | *ypt1-1 ATG39-3xHA* | NSY55 *ATG39-3xHA::KAN* | This study |
| NSY2011 | *ATG39-3xHA pep4∆ prb1∆* | NSY1676 *ATG39-3xHA::KAN* | This study |
| NSY2012 | *ypt1-1 ATG39-3xHA pep4∆ prb1∆* | NSY1678 *ATG39-3xHA::KAN* | This study |
| NSY1528 | TN124 | *Mat****a*** *leu2-3,112 trp1 ura3-52 pho8::pho8∆60 pho13∆::LEU2* | (Noda et al., 1995) |
| NSY1531 | TN124 *atg11∆* | NSY1528 *atg11Δ::KAN* | (Lipatova et al., 2012) |
| NSY2025 | *doa10∆* | NSY825 *doa10Δ::KAN* | This study |
| NSY2026 | *atg11Δ doa10∆* | NSY1510 *doa10Δ::HYGRO* | This study |
| NSY1922 | *trs85∆* | NSY825 *trs85Δ::HYGRO* | (Lipatova et al., 2012) |
| NSY2027 | *trs85Δ doa10∆* | NSY2025 *trs85Δ::HYGRO* | This study |
| NSY1641 | *atg9∆* | NSY825 *atg9Δ::KAN* | (Lipatova et al., 2012) |
| NSY2028 | *atg9Δ doa10∆* | NSY1641 *doa10Δ::HYGRO* | This study |
| NSY2029 | *atg39∆ atg40Δ doa10∆* | NSY2004 *doa10∆::HYGRO* | This study |
| NSY2031 | *trs85∆ hac1∆* | NSY1922 *hac1∆::NAT* | This study |
| NSY2032 | *trs85Δ doa10∆ hac1∆* | NSY2027 *hac1∆::NAT* | This study |
| NSY2013 | NSY825 *ATG40-3xHA* | NSY825 *ATG40-3xHA::NAT* | This study |
| NSY2014 | *atg1∆ ATG40-3xHA* | NSY1567 *ATG40-3xHA::NAT* | This study |
| NSY2015 | NSY825 *ATG39-3xHA* | NSY825 *ATG39-3xHA::NAT* | This study |
| NSY2016 | *atg1∆ ATG39-3xHA* | NSY1567 *ATG39-3xHA::NAT* | This study |
| NSY1962 | MHY8293 | *Mat****α*** *leu2-3,112 ura3-52 his3Δ200 lys2-801trp1-1 TRP1::Deg1-F-Vam12-yEGFP* | Zattas D. and Hochstrasser M. |
| NSY2017 | MHY8293 *doa10∆* | NSY1962 *doa10Δ::KAN* | This study |
| NSY2018 | MHY8293 *trs85∆* | NSY1962 *trs85Δ::HYGRO* | This study |
| NSY2019 | MHY8293 *atg11∆* | NSY1962 *atg11Δ::HYGRO* | This study |
| NSY2020 | MHY8293 *atg9∆* | NSY1962 *atg9Δ::HYGRO* | This study |
| NSY2021 | MHY8293 *doa10∆ trs85∆* | NSY2018 *doa10Δ::KAN* | This study |
| NSY2022 | MHY8293 *doa10∆ atg11∆* | NSY2019 *doa10Δ::KAN* | This study |
| NSY2023 | MHY8293 *doa10∆ atg9∆* | NSY2020 *doa10Δ::KAN* | This study |
| NSY2024 | MHY8293 *doa10∆ atg39∆ atg40∆* | NSY2030 *doa10Δ::KAN* | This study |
| NSY2030 | MHY8293 *atg39∆ atg40∆* | NSY1962 *atg40Δ::HYGRO atg39Δ::NAT* | This study |
| NSY2033 | *RTN1-mCherry* | NSY128 *RTN1-mCherry::KAN* | This study |
| NSY2034 | *ypt1-1 RTN1-mCherry* | NSY55 *RTN1-mCherry::KAN* | This study |
| NSY2044 | *hrd1∆* | NSY825 *hrd1∆::NAT* | This study |
| NSY2045 | *doa10∆ hrd1∆* | NSY2025 *hrd1∆::NAT* | This study |
| NSY2046 | *trs85∆ hrd1∆* | NSY1922 *hrd1∆::NAT* | This study |
| NSY2047 | *doa10∆ trs85∆ hrd1∆* | NSY2027 *hrd1∆::NAT* | This study |
| NSY2048 | *doa10∆* | NSY825 *doa10Δ::NAT* | This study |
| NSY2049 | *atg5Δ doa10∆* | NSY1893 *doa10Δ::NAT* | This study |

**References:**

1. Brachmann C.B., Davies A., Cost G.J., Caputo E., Li J., Hieter P., Boeke J.D. Designer deletion strains derived from Saccharomyces cerevisiae S288C: a useful set of strains and plasmids for PCR-mediated gene disruption and other applications. Yeast 14(2):115-32, 1998.

2. Lipatova Z., Belogortseva N., Zhang X.Q., Taussig D., Segev N. Regulation of selective autophagy onset by Ypt/Rab GTPase module. Proc. Natl. Acad. Sci. USA 109(18):6981-6, 2012.

3. Lipatova Z., and Segev N. A role for macro-ER-phagy in ER quality control. PLoS Genetics 11(7):e1005390, 2015.

4. Noda T., Matsuura A., Wada Y. Novel system for monitoring autophagy in the yeast *Saccharomyces cerevisiae*. Biochem. Biophys. Res. Commun. 210:126-132, 1995.
