# Supplementary material for: Characterization of constitutive ER-phagy of excess membrane proteins
Source: PLoS Genet. 2020 Dec 4;16(12):e1009255. doi: 10.1371/journal.pgen.1009255 (PMC7744050; doi:10.1371/journal.pgen.1009255)
Supplement: S2 Table — (DOCX) [file pgen.1009255.s008.docx]

**S2 Table. Plasmids used in this study.**

| **Plasmid** | **Alias** | **Genotype** | **Source** |
| --- | --- | --- | --- |
| pNS180 | pRS425 | 2µ, *LEU2*, Amp^r^ | (Sikorski and Hieter, 1989) |
| pNS1407 | pRS425-GFP-*SNC1*-PEM | 2µ, *LEU2, TPI* promoter-GFP-*SNC1*-PEM | (Lipatova et al, 2013) |
| pNS1254 | pJC104 | 2µ, *URA3*, 4xUPRE1-crippled *CYC1* promoter-*lacZ* | (Cox et al., 1993) |
| pNS1191 | pFA6a-3xHA-*KanMX6* | *KanMX6*, Amp^r^ | (Longtine et al, 1998) |
| pNS584 | pAG32 | *hphMX,* Amp^r^ | (Goldstein and McCusker, 1999) |
| pNS583 | pAG25 | *natMX4,* Amp^r^ | (Goldstein and McCusker, 1999) |
| pNS254 | pFA6a-GFP(S65T)-*KanMX6* | GFP-*KanMX6*, Amp^r^ | (Bahler et al., 1998) |
| pNS1320 | pBS34 | mCherry-*KanMX6,* Amp^r^ | (Hailey et al., 2002) |
| pNS243 | pRS313 | *CEN*, *HIS3*, Amp^r^ | (Sikorski and Hieter, 1989) |
| pNS1507 | pRS425-Snq2-yEGFP | 2µ, *LEU2, ADH1* promoter-Snq2-YEGFP-CYC1 terminator in pRS425 | (Lipatova and Segev, 2015) |
| pNS1649 | pRS313-*RTN1*-GFP | *CEN*, HIS*3*, *RTN1*-GFP-*ADH1* terminator | This study |
| pNS1650 | pRS313-*NOP1-GFP* | *CEN*, *HIS3*, *NOP1*-GFP-*ADH1* terminator | This study |

**References:**

1. Bahler J., Wu J.Q., Longtine M.S., Shah N.G., McKenzie A., 3rd, Steever A.B., Walsh A., Philippsen P., Pringle J.R. Heterologous modules for efficient and versatile PCR-based gene targeting in Schizosaccharomyces pombe. Yeast 14(10):943-51, 1998.

2. Cox, J.S., Shamu, C.E., and Walter, P. Transcriptional induction of genes encoding endoplasmic reticulum resident proteins requires a transmembrane protein kinase. Cell 73:1197-1206, 1993.

3. Goldstein A.L., McCusker J.H. Three new dominant drug resistance cassettes for gene disruption in Saccharomyces cerevisiae. Yeast 15(14):1541-53, 1999.

4. Hailey D.W., Davis T.N., Muller E.G. Fluorescence resonance energy transfer using color variants of green fluorescent protein. Methods Enzymol. 351:34-49, 2002.

4. Lipatova Z., and Segev N. A role for macro-ER-phagy in ER quality control. PLoS Genetics 11(7):e1005390, 2015.

5. Lipatova Z., Shah, A.H., Kim, J.J., Mulholland, J.W., and Segev, N. Regulation of ER-phagy by a Ypt/Rab GTPase module. Mol. Biol. Cell 24:3133-3144, 2013.

6. Longtine M.S., McKenzie A., 3rd, Demarini D.J., Shah N.G., Walsh A., Brachat A., Philippsen P., Pringle J.R. Additional modules for versatile and economical PCR-based gene deletion and modification in S. cerevisiae. Yeast 14(10):953-961, 1998.

7. Sikorski, R.S., and Hieter, P. A system of shuttle vectors and yeast host strains designed for efficient manipulation of DNA in Saccharomyces cerevisiae. Genetics 122:19-27, 1989.
